# Murine Xenograft Models as Preclinical Tools in Endometrial Cancer Research

**DOI:** 10.3390/cancers16233994

**Published:** 2024-11-28

**Authors:** Merve Yildiz, Andrea Romano, Sofia Xanthoulea

**Affiliations:** 1 GROW—Research Institute for Oncology & Reproduction, Maastricht University, 6229 ER Maastricht, The Netherlands; merve.yildiz@maastrichtuniversity.nl (M.Y.); a.romano@maastrichtuniversity.nl (A.R.); 2 Department of Obstetrics and Gynaecology, Maastricht University Medical Centre, 6229 HX Maastricht, The Netherlands

**Keywords:** xenografts, endometrial cancer, orthotopic, organoids, precision medicine

## Abstract

Murine xenograft models are valuable and increasingly used preclinical tools in cancer research to understand disease pathogenesis and guide treatment options. The aim of this narrative review is to summarize the studies that employed mouse xenograft models, using cell lines, patient-derived tumors, or organoids, in endometrial cancer (EC) research, detailing their methodology and main findings. We identified 27 articles reporting on heterotopic EC xenografts, including subcutaneous, subrenal capsule, intraperitoneal, and retro-orbital models, and 18 articles using orthotopic xenografts. Subcutaneous xenografts generated using either cell lines or patient tumors have been widely used; however, their low engraftment rates and the inability to recapitulate main clinical features such as metastases limit their translational value. Subrenal capsule models showed improved engraftment rates compared to subcutaneous models, but tumors exhibited slower and constrained tumor growth. Orthotopic models are technically more challenging to generate and monitor, but tumor growth occurs in a relevant microenvironment and EC ortho-xenografts exhibit high engraftment rates and metastases to clinically relevant sites. Cell line-based xenograft (CDX) models are attractive tools because they are convenient, easy to use, and amenable to genetic modifications, making them suitable for proof-of-concept approaches and large-scale studies. EC xenografts developed from patient tumors (PDTXs) are more labor/cost-intensive for their establishment but can capture the genetic and molecular heterogeneity within and across histologic subtypes and can inform personalized patient treatment. EC organoid-based xenograft (PDOX) models combine the advantages of both CDXs and PDTXs since they are more time- and cost-effective, faithfully maintain tumor characteristics and therapeutic responses, and can be genetically modified. Despite substantial progress in EC management, there are still several unmet needs. Efficient targeted treatments are currently indicated only for a small subgroup of patients, while women with recurrent or advanced-stage EC have very few therapeutic options and their prognosis remains unfavorable. Novel (targeted) drugs, combinational regimens and tools to predict the real drug response in patients are urgently needed. Xenograft models are expected to inform about disease mechanisms and to help identify novel therapeutic options and suitable target patients.

## Simple Summary

With both prevalence and mortality increasing, endometrial cancer (EC) is the most common gynecological cancer in high-income countries. Despite significant advances in EC research and management, there are still unmet needs. Patients suffering from advanced-stage and recurrent EC lack treatment options and have poor prognosis. Robust preclinical models are crucial in the development of novel therapies, and xenograft models have recently gained increasing interest in drug discovery and precision medicine. A clear overview of all developed EC mouse xenograft models is currently lacking. The aim of this review is to summarize these studies, reporting on their methodology and main findings. The different models are grouped based on the source material used to generate the xenografts, i.e., cell lines, patient-derived tumors, or patient-derived tumor organoids, and on the location of tumor formation, i.e., heterotopic or orthotopic. Finally, the advantages and disadvantages of the different tumor source material and xenograft locations, as well as some considerations on the translational potential, limitations, and future directions of EC xenograft models, are discussed.

## 1. Introduction

Endometrial carcinoma (EC) is a type of cancer that develops in the inner lining of the uterus called the endometrium, and with approximately 420,000 new cases and 98,000 deaths in 2022, it ranks 6th in incidence and 13th in mortality among women’s cancers worldwide [1]. Risk factors for developing EC are conditions leading to unopposed estrogen exposure of the endometrium like obesity, nulliparity, early-onset menarche, late menopause, diabetes, and older age. Overall incidence and mortality have risen disproportionate to other cancers during recent decades, reflecting a rise in the prevalence of risk factors, mainly aging and obesity [2]. Of the 20 most common tumor types, EC has the strongest link with obesity, with every 5 kg/m^2^ increase in BMI associated with a 54% higher risk of cancer [3]. EC is the most common gynecologic cancer in high-income countries and, in some communities, is already more common than breast cancer and leads to the death of more women than lung cancer [4,5]. Unfortunately, the diet and lifestyle of high-income countries are spreading worldwide, also increasing the incidence of EC in low-resource countries and leading to predictions of an increase in EC deaths of 90% by 2050 [6].

EC affects mainly post-menopausal women, with an average age at diagnosis of 60 years and the mainstay of treatment comprising the surgical removal of the cervix, uterus, ovaries, and fallopian tubes. Adjuvant treatment greatly depends on the prognostic risk group and generally involves chemotherapy and/or radiation therapy. Risk group classification has improved since the introduction of molecular classification, which categorizes tumors into four groups according to their molecular profile. In clinical practice, such classification relies on results of tumor immunohistochemistry for mismatch repair (MMR) proteins and p53 (the protein product of tumor suppressor gene *TP53*), as well as sequencing for hotspot mutations of the gene encoding for the enzyme DNA polymerase epsilon (*POLE*). The four molecular subgroups are (1) ultramutated tumors characterized by *POLE* hotspot mutations, with excellent prognosis; (2) hypermutated tumors characterized by mismatch repair deficiency (MMRd), resulting in microsatellite instability (MSI), with good/intermediate prognosis; (3) tumors with no specific molecular profile (NSMP) or copy number low (p53 wild-type), with intermediate prognosis; (4) copy number-high (CNH) tumors characterized by *TP53* mutations (p53 abn), with poor prognosis [7]. The ESGO/ESTRO/ESMO guidelines from 2022 have incorporated this molecular classification to aid decision-making regarding the use of chemo/radiotherapy as adjuvant treatment [8]. The management of advanced unresectable disease, multifocal recurrent, and metastatic EC is mainly palliative and requires a multi-disciplinary approach, including patient preference. During the last five years, the use of targeted therapy for EC has been explored. These treatments include hormonal therapy, immune checkpoint inhibitors, cellular pathway inhibitors, vascular endothelial growth factor (VEGF) inhibitors, DNA repair pathway inhibitors, and combination therapies [2,7,9]. Results from large clinical trials are encouraging and showed the effectiveness of immune checkpoint inhibitors as mono- or combinational therapy for advanced-stage EC. Additionally, the use of these drugs, initially restricted to the MMRd group, has recently been approved in association with VEGF inhibitors as second-line treatment for advanced-stage ECs [10,11]. Despite this progress, effective treatments for p53 abn and NSMP groups are still lacking. Women in the NSMP subcategory, representing the largest group of patients, present with heterogenous tumor characteristics and prognosis, and they need better personalized and targeted options. Moreover, women with EC are frequently elderly and often present important comorbidities and should therefore be indicated for mild/nonaggressive therapeutic options.

Relevant in vivo pre-clinical models are invaluable tools to progress our understanding on EC progression, metastases, and response to drugs, and in recent years, efforts have intensified for the development of such models. The aim of the current review is to provide a detailed overview of publications that have used mouse xenograft models in EC research. Articles were retrieved either from the National Library of Medicine (PubMed) or through references, with no restrictions on the year of publication. We describe these studies and discuss their main findings, compare the different locations for xenografting (heterotopic vs. orthotopic) and the use of cell lines vs. patient-derived material, and finally reflect on the translational value and the potential risks and possibilities of EC xenograft models.

## 2. Mouse Endometrial Cancer Xenograft Models

Xenografts refer to cells, tissues, or organs that derive from a donor of one species and are engrafted to a recipient of another species. Heterotopic xenograft models are generated when engraftment occurs in an anatomical location different from that of the original tissue, while in orthotopic xenografts, the same anatomical location is used, e.g., the uterus in the case of EC. Mouse EC xenograft models derive from the (heterotopic or orthotopic) engraftment of human EC cell lines, organoids, or surgically removed patient-derived tumor tissue into immunocompromised mice. Cell line-derived xenografts (CDXs) present several disadvantages compared to patient-derived tumor xenografts (PDTXs) and patient-derived organoid-based xenografts (PDOXs). Since most cell lines have lived for hundreds of generations in cell culture conditions, their original genetic make-up and tumor characteristics have most likely been altered. Moreover, CDXs are largely homogenous and reflect neither the intra-tumor heterogeneity of cancer cells, exhibiting different molecular and phenotypical profiles, nor the inter-patient tumor heterogeneity consisting of different histological and molecular subtypes. In addition, 2D cell cultures lack the complex multicellular 3D environment and the dynamic cell–cell interactions occurring in the tumor microenvironment. Due to these limitations, treatments developed with these models often fail to be translated into clinical settings, raising questions regarding their clinical predictive value. Contrarily, PDTXs are able to represent the heterogeneity and gene expression profile of the original tumor and can recapitulate the patient-specific responses to chemotherapy and targeted therapies, thus being the most clinically relevant in vivo cancer model to date [12]. Therefore, in recent years, several institutions and pharma and biotech companies are creating PDTX repositories to replace human cancer cell lines and meet the needs for more relevant pre-clinical models for cancer research. However, like any other model system, PDTXs have limitations: PDTXs (and xenograft models in general) are developed in immunocompromised mice, thus posing a challenge to immuno-oncology research. In different PDTXs, mouse-specific evolution during in vivo passaging has been reported, with an accumulation of genetic alterations that distances the xenografts from the original tumors and confers a more aggressive phenotype on them [13,14]. This should be considered when using PDTXs as “avatar” models for personalized medicine or for drug screening purposes, and it suggests studies be conducted using low-passage xenografts. PDTXs show a gradual loss of the human tumor microenvironment with, for example, tumor-derived stromal fibroblasts being slowly replaced by mouse-derived ones, thus interfering with anticancer therapies targeting the tumor stromal compartment. Individual PDTX models are usually established using a single piece of tumor tissue which, however, might not be representative of the entire tumor due to high intratumor heterogeneity. The establishment of PDTXs requires a substantial investment in time (several months) and resources. For most patients, it is unrealistic to wait for the model of their tumor to be established and undergo drug testing before they can benefit from the screening results. Recently, different studies have used patient-derived organoid-based xenograft models (PDOXs) and argue that these may better retain the features of parental tumors and thus serve as more reliable, faster, and more convenient preclinical cancer models compared to CDXs and PDTXs [15,16,17,18,19,20,21,22,23,24].

## 3. Heterotopic Xenografts

Heterotopic xenografts have been widely used in EC research to study disease progression and response to treatments. These heterotopic xenografts derive from either human EC cell lines (CDXs), from patient tumors (PDTXs), or from organoids generated from tumor tissue and subsequently transplanted to mice (PDOXs). The preferred site of xenograft engraftment in most studies is the subcutaneous tissue, with some studies establishing xenografts in the subrenal capsule, intraperitoneally, or retro-orbitally (used as an EC–lung metastasis model). In Table 1, we summarize these publications and discuss them below. In Supplementary Table S1, we report the details of the cell lines and their resource identifiers (RRIDs) used for subcutaneous xenograft generation in the different studies.

### 3.1. Subcutaneous Models

The first studies to establish subcutaneous EC xenografts used EC cell lines (CDXs) and tested the efficacy of different targeted therapies on tumor growth. To study the role of epigenetics and the effects of histone deacetylase inhibitors (HDACs) on endometrial cancer cells when combined with traditionally used cytotoxic agents, Dowdy et al. generated a xenograft model through the flank injection of endometrial serous adenocarcinoma Ark2 cells in nude mice. They found that the HDAC inhibitor trichostatin A (TSA), together with paclitaxel, synergistically inhibited the proliferation of serous endometrial cancer cells and activated the apoptotic cascade, resulting in a 50% reduction in tumor weight compared with either agent alone [25]. Takahashi et al. used the endometrial adenocarcinoma HEC-1A cell line to model a series of EC clinical conditions: They injected cells subcutaneously in nude mice to measure tumor growth; intraperitoneally, to model a peritoneal dissemination model; within the uterus, to develop a lymph node metastases model; in the tail vein, to develop a lung metastases model; and in the peritoneal cavity and tail vein, to develop a systemic metastases model. Mice were treated with an anti-EGFR monoclonal antibody (cetuximab). The authors state that tumor growth, peritoneal dissemination and ascites volume, and lymph node and lung metastasis were inhibited in the treated group. Moreover, survival in the mouse model of systemic metastases was significantly prolonged in the cetuximab treated mice [26]. Pant et al. used endometrial adenocarcinoma Ishikawa cells to establish subcutaneous xenografts in nude mice and showed that inhibition of the AKT pathway in combination with progestin decreases cancer cell proliferation, increases apoptosis, and inhibits tumor growth. Therefore, AKT pathway inhibition could improve the efficacy of progestins in the treatment of endometrial cancer [27]. In two studies, Packer et al. used xenografts of FGFR2 mutant EC cell lines AN3CA and JHUEM2, established subcutaneously in non-obese diabetic (NOD) and severe combined immunodeficient (SCID) or NOD SCID gamma (NSG) mice. In one study from 2017, they examined the efficacy of the pan-FGFR inhibitor BGJ398 with pan-PI3K inhibitor GDC-0941, as well as the p110a-selective inhibitor BYL719. BGJ398 alone resulted in significantly delayed tumor growth. Combinatory treatment with BGJ398+GDC-0941 and BGJ398+BYL719 resulted in a marked inhibition of tumor growth in both AN3CA and JHUEM2 xenograft models, compared with the BGJ398-treated groups, indicating a synergistic effect [28]. In a second study from 2019, the authors tested the combination of Bcl-2 inhibitor ABT263 with FGFR inhibitor BGJ398 in AN3CA subcutaneous xenografts. The combination of BGJ398+ABT263 significantly improved the antitumor response to BGJ398 alone and caused an 11-fold increase in caspase-3 cleavage compared to controls. The authors showed that FGFR inhibition induces mitochondrial-dependent cell death, and when combined with BH3 mimetics (such as ABT263), it leads to enhanced cell death, likely through caspase activation [29]. To dissect the resistance of advanced uterine carcinomas to another targeted drug, the multi-kinase inhibitor sorafenib, Eritja et al. generated CDX tumors and an EC–lung metastatic assay by injecting MFE-296 adenocarcinoma cells into the subcutaneous flank and retro-orbital space, respectively, of SCID mice. Moreover, they generated primary EC ortho-xenografts of grade-I to -III tumors (further discussed below). Using these models, they could show that targeting autophagy enhances the cytotoxic and tumor suppressive action of sorafenib and suppresses pulmonary metastases [30].

Subsequent studies used subcutaneous PDTXs (in some studies in parallel with tumor subtype-matched CDXs), to test novel single agents or combination therapies on xenograft growth. In a study from 2014, Groeneweg et al. used either patient-derived uterine serous carcinoma (USC) cells or USC cell lines to establish subcutaneous xenografts in NOD/SCID mice and test the efficacy of gamma secretase-mediated Notch pathway inhibition on tumor growth. USC tissue was first enzymatically processed and then depleted of endothelial and hematopoietic cells. Treatment with gamma secretase inhibitor as monotherapy or in combination with standard paclitaxel–carboplatin (PTX-CBP) therapy moderately reduced tumor growth in one out of two USC xenografts [31]. In a second study and by using xenografts from either USC cell lines or from USC patients, the same authors investigated the effectiveness of human epidermal growth factor receptor 2 (HER2) inhibition using the HER2 inhibitors lapatinib and trastuzumab. The authors showed that lapatinib as a single agent, or in combination with trastuzumab, induced significant growth-inhibitory effects only in tumors harboring *HER2* gene amplification. In the non-amplified-*HER2* tumor xenografts, a complete lack of response to any administered therapy was seen. They concluded that *HER2* gene amplification might be used as a biomarker for the response to HER2 inhibition in USCs [32]. Bradford et al. investigated the anti-tumor activity of the pan-class I PI3K inhibitor NVP BKM-120 (BKM) as a single agent and in combination with standard cytotoxic chemotherapy (PTX-CBP) in NOD/SCID mice bearing subcutaneous xenografts of either a *PIK3CA* mutant or *PIK3CA* wild-type endometrial tumors (grade 2 or 3 and of endometrioid, serous (USC) and carcinosarcoma histological subtypes). Patient tumors were initially propagated subcutaneously by the injection of single-cell suspensions that were depleted of hematologic components, and formed primary xenografts were excised, depleted of mouse H-2Kd+ cells, and subcutaneously injected into new recipient NOD/SCID mice. Independent of *PIK3CA* gene mutation, BKM treatment induced both the inhibition of the PI3K/AKT/mTOR pathway and initial tumor growth in xenografts. However, a pattern of resistance emerged which could be partly mitigated in some xenografts by the addition of chemotherapy [33]. Dosil et al. established subcutaneous tumors in nude mice using endometrial adenocarcinoma HEC-1A or MFE-296 cells and PDTXs from an endometrioid endometrial cancer, to examine the effect of cyclin D–CDK4/6 inhibition by palbociclib (PD-332991) on endometrial tumors driven by *Pten* deficiency. Palbociclib treatment led to a significant reduction in tumor growth, revealing an important role for CDK4/6 activity in the development of endometrial malignancies [34]. Yu et al. assessed the combined effect of ARQ 092, a pan-AKT inhibitor, and ARQ 087, a pan-FGFR inhibitor, in nude mice using the AN3CA endometrial adenocarcinoma cell line and patient-derived tumors bearing PIK3CA and FGFR mutations. Enhanced antitumor activity when ARQ 092 and ARQ 087 were combined was observed [35]. Studying another AKT pathway inhibitor, Felip et at. generated subcutaneous CDXs in nude mice using the HEC-1A cell line and PDTXs from a grade-2 endometrioid and a grade-3 serous ECs (both bearing PI3KCA mutations), to test the efficacy of the PI3K/AKT/mTOR pathway inhibitor ABTL0812. In both the CDX and PDTX models, ABTL0812 as monotherapy inhibited tumor progression with a comparable efficacy to the standard first-line chemotherapy treatment (PTX-CBP). The combination of ABTL0812+PTX-CBP treatment showed an increased efficacy in inhibiting tumor growth compared to individual treatments [36]. 

More recent studies provided a detailed characterization of PDTXs at the histologic, molecular, genomic, and transcriptomic levels and determined their resemblance to the original tumors. Depreeuw et al. were the first to characterize a panel of PDTX models from the major histological and molecular subtypes of EC (primary, metastatic, and recurrent type-I and type-II EC), by the subcutaneous implantation (in the flanks or interscapular region) of tumor tissue in immunocompromised nude mice. Of the 40 fresh primary, metastatic, and/or recurrent EC samples, 24 successfully engrafted (overall 60% success rate) with an F0-F1 engraftment time between 1.5 and 9 months. PDTXs were shown to closely resemble the original tumors in terms of histology and genomic features. Whole exome sequencing (WES) and copy number variation (CNV) analyses of four models indicated that most mutations were common between primary tumors and xenografts. Estrogen (ER) and progesterone (PGR) receptor staining showed good similarities between patient and xenografted tumors although, in general, PGR expression was reduced in xenografts. Moreover, staining for stromal cells showed that the human stromal compartment is replaced by murine stroma after engraftment. In addition, a significant enrichment in MSI and *POLE* mutations was found among the engrafted tumors compared to the non-engrafted ones, suggesting a growth advantage for such hypermutated tumors in mice and indicating the importance of testing endometrioid EC PDTX models for MSI and *POLE* mutations, especially when these models are used for the evaluation of targeted therapies. Combination treatment with a dual pan-PI3K/mTOR inhibitor and a MEK1/2 inhibitor showed the stabilization of tumor growth in one model [37]. Two studies developed PDTXs models from uterine sarcomas (USs) and carcinosarcomas (CSs), which are rare but aggressive cancers with a poor prognosis since they show little response to chemotherapy regimens. Cuppens et al. established and characterized 13 subcutaneous PDTXs derived from different human uterine sarcoma (US) and carcinosarcoma (CS) patient samples. For leiomyosarcomas (LMSs, the most common US subtype), a success rate of 77% was observed, while for CS, it was 29%. No differences in success rates were observed between primary and recurrent or early- and late-stage tumors. The analysis of copy number profiles obtained by whole genome sequencing (WGS) showed a median similarity of 86% between the original and xenografted tumors. While, in general, all models were histologically stable, some xenografts showed numerous changes in their genomic and/or transcriptomic profiles compared to their corresponding human tumors, which should be considered when performing preclinical studies [38]. In addition, from the same research group, the authors used subcutaneous xenografts of uterine leiomyosarcomas from five patients and showed that dual PI3K/mTOR inhibition exhibits a strong reduction in tumor growth [39]. Shin et al. subcutaneously transplanted tumor tissue from 32 uterine cancers (22 endometrioid, 5 serous, 1 clear cell carcinoma, 3 carcinosarcomas, and 1 stromal sarcoma) in nude mice and observed an engraftment rate of 45%, 80%, 100%, 100%, and 0%, respectively, a mean time for tumor formation of 6 months, and higher engraftment and tumor growth rates by subsequent passages (P2 and P3). There was an association between the PDTX success rate and tumor grade (*p* = 0.0025), but not with other clinicopathological characteristics, and no association between PDTX engraftment or growth rate and patient survival [40]. Bonazzi et al. generated PDTX models representing all four molecular subtypes of EC, including varying histology types (carcinosarcoma, endometrioid, mixed endometrioid and serous, and mixed endometrioid and clear cell) and stages (IA to IIIB), by inserting a tumor piece in the interscapular region of NSG mice. Successful engraftment rates were only obtained for freshly implanted tumors of histological grades 2 and 3 (33% and 61%, respectively), while no grade-1 tumors engrafted (0/8). Engraftment was less successful after storage at −80 °C or overnight at 4 °C. The process of PDTX engraftment selected for more aggressive subtypes, and engraftment success was significantly associated with shorter disease specific survival (*p* < 0.02). Although mutational heterogeneity seemed to be more frequent in the MMRd molecular subtype PDTXs, the overall mutational profiles were very similar between the primary and matched PDTX samples at different transplantation passage numbers, and the accumulation of PDTX-specific copy number alterations (CNA) events was not observed. Therefore, PDTX-specific evolution was minimal, and PDTXs reliably represented the driver events and molecular subtypes of the primary tumors. By combining genomic characterization and in vivo treatments, the authors also showed that the PARP inhibitor talazoparib had tumor growth inhibition activity in CN-high/p53mut EC PDTXs [41]. Villafranca-Magdalena et al. engrafted tissue samples from high-risk and recurrent EC patients with molecular profiles of endometrioid/MSI or serous/HCN subcutaneously in nude mice, and by using immunohistochemistry and WES data, they determined a good resemblance between the PDTXs and the original tumors. The authors observed clear differences between the MSI and HCN groups regarding molecular alterations, with each tumor type having a distinct genetic profile that matched the genomic profile described by the TCGA for each EC subtype. These differences were reflected in the corresponding PDTXs, with different patterns of preservation of single-nucleotide variants (SNVs) and copy number variants (CNVs) in the two subtypes [42]. In a recent study, Imai et al. aimed to study the alterations in the molecular properties of PDTXs when passaged up to eight times subcutaneously in NOG or NSG mice. They used tumors from nine endometrioid carcinomas (including one mixed endometrioid/serous) and three carcinosarcomas. Engraftment rates were 33% and 100% for the endometrioid carcinoma and carcinosarcoma samples, respectively. PDTXs from carcinosarcomas, which consisted of both carcinoma and sarcomatous components in the parental tumors, were predominantly composed of sarcomatous components in the PDTXs in generation 1 (G1) or later, possibly due to the positive selection in PDTXs of the aggressive sarcomatous components. This should be a point of consideration when using PDTXs from carcinosarcomas to test therapeutic targets. In contrast, PDTXs from endometrioid cancers maintained their morphological characteristics from G1 to G8. Authors also noticed alterations in the immunohistochemical characteristics of ER, PTEN, PAX8, and PAX2 in the PDTXs compared to the original tumors, probably because of the dedifferentiation or EMT of tumor cells in PDTXs. In addition, some of the representative genomic/phenotypic alterations that are used as the basis for the molecular classification of endometrial cancer were affected during the establishment and passaging of PDTXs, indicating that genomic and phenotypic similarities between primary tumors and PDTXs need to be verified when using PDTXs for drug screening/preclinical studies [43]. Finally, Sengal et al. used PDXs implanted subcutaneously in NSG mice to test the therapeutic efficacy of FGFR inhibitors BGJ398 or Pemigatinib. PDXs with high/moderate FGFR2c isoform expression showed significant tumor growth inhibition following treatment with FGFR inhibitors and significantly prolonged survival in four out of five models. The addition of cisplatin to Pemigatinib showed benefit in two PDX models representing p53abn EC, indicating that FGFRi might be chemosensitizing to only a subset of patients with p53abn EC. BGJ398 treatment resulted in a significant reduction in cancer cell proliferation and microvascular density with the reduced expression of several angiogenic growth factors like VEGFA, CTGF, and GDF15, as well as a significant reduction in pro-tumor CD206+ M2 polarized macrophages, indicating a central role for FGFR inhibition in the modulation of the immune TME [44].

Using patient-derived organoids, in a study by Pauli et al., subcutaneous organoid-derived xenografts were generated. Organoids were initially generated from two late-stage uterine cancers, a stage-IIIB uterine carcinosarcoma and a stage-IIIC2 endometrial adenocarcinoma, and 10^6^ cells of tumor organoids were injected subcutaneously in the flanks of nude mice to generate patient-derived organoid xenografts (PDOXs). Tumor organoid and PDOX histology showed conservation of the histopathologic features of the native tumors, while allele-specific copy number analysis showed a median of 86% concordance between organoids and PDOXs to the native tumor tissues. Similarly, SNV analysis showed excellent concordance between native tumors and matching organoids and PDOXs. The authors used PDOXs to validate high-throughput drug screen results obtained from ex vivo 2D and 3D organoid cultures and showed a good concordance of responses between in vivo PDOXs and ex vivo organoid models [22].

### 3.2. Subrenal Capsule Models

In addition to the above subcutaneous xenograft models, a number of studies established EC xenografts in the subrenal capsule of immunocompromised mice. This location provides high levels of blood and lymph flow rates and positive interstitial fluid pressure, ensuring an abundant supply of nutrients, hormones, growth factors, and oxygen to transplanted tissues. Consequently, xenograft engraftment rates are generally better compared to subcutaneous grafting [45]. Zhu et al. compared the establishment rates of PDTXs in the two locations using 18 primary high-risk endometrial cancers (10 cases of high-grade EC, 6 cases of serous carcinoma, 1 case of clear cell carcinoma, and 1 case of carcinosarcoma). Success rates of 50% and 62.5% were achieved for the subcutaneous and subrenal capsule engraftment of PDTXs, respectively, but this difference was not significant (*p* = 0.464). The mean time for tumor formation after subcutaneous grafting was 4–5 weeks, and 8–9 weeks for the subrenal capsule, with faster growth rates in subsequent passages. WES and RNA sequencing of original tumors and passage-3 PDTXs indicated similar but not identical profiles [46]. Press et al. transplanted a high-grade uterine leiomyosarcoma under the renal capsule of NOD/SCID mice. A comparison between the primary tumor and the corresponding generation-3 xenograft by CGH array and immunohistochemistry showed genetic and phenotypic concordance, indicating genetic and phenotypic stability during serial transplantations [47]. In a study by Unno et al., tumor tissue fragments from different types of endometrial cancers like uterine serous carcinoma (USC), uterine clear cell carcinoma (UCCC), malignant mixed Mullerian tumor (MMMT), and endometrioid endometrial carcinoma (EEC) were transplanted under the renal capsule of NSG mice. Among these, USC, MMMT, and EEC established and grew. Engraftment take rates varied between 6% and 97% and depended on the type of tumor and the presence or not of estradiol. Only one of the two EECs showed dependency on E2 for engraftment, while other tumor subtypes did not. Tumors were subsequently characterized using various markers for tumor subtype, EMT, and steroid receptors, and they were shown to retain the characteristics of the original tumor. In addition, the authors examined the metastatic potential, which varied between the grafts and depended on their individual tumor characteristics. USC tumors showed no kidney invasion or local metastases, while MMMT and EEC tumors showed local invasion to peritoneal organs and liver metastases [48]. From the same research group, Winder et al. engrafted three lines from the above-established xenografts (uterine serous, endometrioid grade 2, endometrioid grade 3) under the renal capsule of NSG mice, and they used the MK2206 AKT inhibitor to test tumor growth and invasion. The tumor volume of all three PDTX lines was significantly reduced in the inhibitor-treated mice, in addition to decreased tumor invasion within the kidney [49]. Jeong et al. generated 33 PDTXs from different histological types and FIGO stages of ECs, by inserting small pieces of tumor tissue both heterotopically in the subrenal capsule and orthotopically into the uterine lumen. They report a subrenal capsule engraftment rate of 50% for endometrial carcinoma, 80% for uterine sarcoma, and 75% for carcinosarcoma, with successful engraftment not associated with the clinicopathological characteristics of the tumors. The engraftment rate was 100% for the orthotopic xenografts. Some of the carcinosarcoma-derived PDTXs exhibited a loss of the epithelial component and prevalence of the mesenchymal part, while one carcinosarcoma model showed only the epithelial component in the PDTX. The authors confirm that human stroma is gradually replaced by murine stroma during in vivo passaging (except for the sarcoma cases). Xenografts maintained histologic stability through different generations, and short tandem repeat (STR) and targeted sequencing analyses revealed that all mutated cancer-related genes were stable during establishment and sub-grafting. The authors also preclinically tested the PI3K inhibitor AZD8835 in two subrenal xenografts, an endometrial clear cell carcinoma harboring a *PTEN* deletion and an endometrioid carcinoma harboring a *PIK3CA* mutation. In both cases, treatment with the inhibitor reduced tumor growth. Interestingly, none of the subrenal PDTXs produced metastatic lesions [50].

### 3.3. Other Models

Injections of tumor cells in other anatomical locations in immunocompromised mice have also been performed (Table 1), and most have been reported above. A recent study by Colon-Otero et al. used an intra-peritoneal injection, in SCID mice, of a suspension of tumor cells derived from biopsies of ER-positive metastatic lesions [51]. Engraftment rates were 55% for grade-1 and-2 endometrioid EC, 100% for high-grade endometrioid EC, and 40% for high-grade serous EC. Across all models, the average time from the tumor injection to the first tumor harvest was 196 days, highlighting one of the limitations of using PDTX models, i.e., the long times needed to complete studies. The tumor diameter and cross-sectional area were measured by transabdominal ultrasound. The ER and histological pattern were preserved across samples. Three EC models were tested for the clinical effectiveness of a combination of the cyclin kinase inhibitor ribociclib and the aromatase inhibitor letrozole. Slower tumor progression and a positive survival effect on the mice were observed in two of three PDTX models.

**Table 1:** Heterotopic xenograft EC models.

	Subcutaneous	Subrenal Capsule	Intra-Peritoneally	Other Locations
**CDXs**	Dowdy et al. *Mol Cancer Ther* (2006) [25]		Takahashi et al. *Int J Oncol* (2009) [26]	Tail vein: Takahashi et al. *Int J Oncol* (2009) [26]
	Takahashi et al. *Int J Oncol* (2009) [26]			Retro-orbital: Eritja et al. *Autophagy* (2017) [30]
	Pant et al. *PLoS One* (2012) [27]			
	Packer et al. *Mol Cancer Ther* (2017) [28]			
	Eritja et al. *Autophagy* (2017) [30]			
	Packer et al. *Mol Oncol* (2019) [29]			
**PDTXs**	Groeneweg et al. *Gynecol Oncol* (2014) [31] *	Press et al. *Gynecologic Oncology* (2008) [47]	Colon-Otero et al. *ESMO Open* (2020) [51]	
	Groeneweg et al. *Clin Cancer Res* (2014) [32] *	Unno et al. *PLoS One* (2014) [48]		
	Bradford et al. *Gynecol Oncol* (2014) [33] #	Winder et al. *Cancer Biol Ther* (2017) [49]		
	Depreeuw et al. *Gynecol Oncol* (2015) [37]	Zhu et al. *Int J Gynecol Cancer* (2018) [46]		
	Dosil et al. *J Pathol* (2017) [34] *	Jeong et al. *Gynecologic Oncology* (2021) [50]		
	Yu et al. *Anticancer Drugs* (2017) [35] *			
	Cuppens et al. *Gynecologic Oncology* (2017) [38]			
	Cuppens et al. *Clinical Cancer Research* (2017) [39]			
	Zhu et al. *Int J Gynecol Cancer* (2018) [46]			
	Felip et al. *Gynecol Oncol* (2019) [36] *			
	Shin et al. *Cancers (Basel)* (2022) [40]			
	Bonazzi et al. *Genome Med* (2022) [41]			
	Villafranca-Magdalena et al. *International Journal of Molecular Sciences* (2022) [42]			
	Imai et al. *Scientific Reports* (2023) [43]			
	Sengal et al. *npj Precision Oncology* (2023) [44]			
**PDOXs**	Pauli et al. *Cancer Discov* (2017) [22]			

* Tumor subtype-matched CDXs generated in parallel experiments. # Initial subcutaneous propagation step of patient tumor.

## 4. Orthotopic Xenografts

Orthotopic xenograft models are clinically more relevant compared to heterotopic models as they more closely mimic the complex tumor microenvironment and metastases seen in patients. This results in a more accurate recapitulation of the human disease, enhancing the predictive value of the models and the clinical significance of the results. In addition, orthotopic models have higher tumor engraftment rates. The drawbacks are the technical difficulty to perform microsurgery in mouse uteri and the difficulty to monitor tumor growth and metastases, which requires adequate imaging techniques. To meet these needs, Haldorsen et al. examined the same mice in parallel by in vivo bioluminescence imaging (BLI), positron emission tomography–computed tomography (PET-CT), and magnetic resonance imaging (MRI) in orthotopic EC models. Ishikawa cells were stably transfected with luciferase and injected into the uterine horn of NSG mice. MRI and PET-CT with ^18^F-FDG and ^18^F-FLT tracers, able to image the different metabolic properties of the tumors, were performed weekly. ^18^F-FLT is taken up by proliferating cells in the S-phase and is thought to precisely indicate the presence of viable tumor cells. ^18^F-FDG is a glucose analogue which accumulates in tissues with increased metabolic activity, and it is not specific for tumor tissue. Metastases (in the ovaries, kidney, spleen, pancreas, liver, lung, the connective tissue surrounding the uterus, adrenal glands, and lymph nodes) were seen in 82% (9/11) of the mice and were detected by the applied imaging methods. The authors also generated an orthotopic PTDX model using a grade-3 EEC. Primary tumor was mechanically disrupted, and cells were injected into the endometrial cavity of four NSG mice for F1 xenograft generation. A cell suspension of F1 tumors was subsequently orthotopically implanted into the next generation (F2) of NSG mice. F1 and F2 tumors were classified as grade-3 EECs and histologically resembled the patient tumor. PET-CT was successfully used to detect PDTX growth [52]. In studies by Haldorsen et al., static PET imaging was used, which acquires a static image at a pre-established time point following injection. Dynamic PET imaging acquires kinetic information by continuous imaging over a pre-defined period immediately after injection, which could have more potential in monitoring tumor progression in preclinical models. In 2021, Espedal et al. used dynamic ^18^F-FDG-PET imaging together with MRI for monitoring tumor growth and chemotherapy response in an orthotopic patient derived organoid xenograft (PDOX) EC model, which is discussed below. Another imaging modality that has been investigated is near-infrared fluorescent (NIRF) optical imaging. This approach uses exogenous fluorescent antibodies that target specific molecular markers to visualize tumor growth. NIRF has some benefits, as there is no need for the genomic introduction of reporter genes into EC cells or radioactive tracers, and the scanning time is shorter compared to PET/CT. However, NIRF imaging is not quantitative and lacks three-dimensional information. Fonnes et al. aimed to investigate a marker that is universally expressed in EC for the targeted NIRF imaging of orthotopic models [53]. The authors selected the epithelial cell adhesion molecule (EpCAM), which was found to be highly expressed in EC cell lines and in the majority of EC primary tumors (150 out of 153). EpCAM was also significantly associated with histological grade, with high expression in 84% of patients with endometrioid EC compared to 64% and 56% of patients with serous EC and carcinosarcomas. To generate the orthotopic models, NSG mice were injected with HEC-1B cells or Ishikawa cells (transfected with luciferase for BLI for parallel imaging). In both CDX models, EpCAM NIRF and BLI demonstrated comparable capacities in detecting primary tumors, while metastatic lesions were better detected with the EpCAM NIRF approach. Moreover, authors used four patient tumor samples (manually dissociated into cell suspensions) to generate orthotopic PDTXs in NSG mice. EpCAM NIRF imaging was compared with ^18^F-FDG-PET/CT imaging. Overall, EpCAM NIRF imaging appeared to be superior in detecting tumors earlier and more distinct. Mice with orthotopic PDTXs from a grade-3 EEC were also treated with paclitaxel or trastuzumab (HER2 inhibitor), and although none inhibited tumor development, EpCAM-NIRF was capable of successfully monitoring uterine tumors. Moreover, Shen et al. [54] used ultrasound scanning to follow tumor growth (discussed below). In Table 2, we summarize the studies that used orthotopic EC xenograft models.

### 4.1. Cell Line-Derived Orthotopic Xenograft Models

Using cells lines, early attempts to generate orthotopic xenografts were made by Kamat et al. [55] and Lee et al. [56] by injecting endometrial adenocarcinoma Ishikawa cells or HEC-1A cells into the uterine horn of athymic nude mice. Both models exhibited a representative natural progression of human EC and could recapitulate the pattern of metastasis seen in patients. Lee et al. aimed to determine the biological activity of an EphA2-targeted antibody drug conjugate that selectively binds to both rodent and human EphA2 receptors. Metastatic spread was detected with bioluminescence imaging (BLI) in the abdominal cavity of mice injected with Ishikawa cells. In mice injected with HEC-1A cells, metastasis was detected in the pelvis, mesentery, omentum, liver, porta hepatis, peri-splenic area, para-aortic lymph nodes, and diaphragm. The EphA2-targeted antibody reduced primary tumor and metastatic spread in both cell line models. Kamat et al. investigated the therapeutic efficacy of targeting vascular endothelial growth factor (VEGF) using bevacizumab alone or in combination with docetaxel. The most prevalent sites of metastatic spread for both cell lines were the peritoneum (>75%), kidney (<10%), mesentery (50–57%), liver (<10%), and lymph nodes (43–63%), detected by BLI. For both cell lines, monotherapy reduced metastatic spread, which was further reduced for the combination treatment. Takahashi et al. aimed to test the effects of a soluble decoy receptor for prostaglandin E_2_ (PGE_2_), as PGE_2_ is hypothesized to enhance the progression of EC. Ishikawa cells were transfected with the decoy receptor and injected in the uterus of ovariectomized mice, with an engraftment rate of approximately 85%. The mean tumor volume was significantly decreased (8-fold) in mice injected with the transfected cells compared to cells transfected with an empty vector, suggesting that the decoy receptor for PGE_2_ can affect tumor cell proliferative status, thereby being a potential candidate for gene therapy [57]. In addition, Takahashi et al. aimed to develop a suitable animal model for evaluating the role of lymph node metastasis in EC [58]. VEGF-C is produced by tumor cells and is suggested to be a significant promoter of lymph node metastasis. The authors transduced HEC-1A cells with a VEGF-C expression plasmid and injected cells into the uterine cavity of nude mice. Eight weeks after injection, most mice developed lymph node metastasis, with increased metastatic rates in VEGF-C-overexpressing mice. Cabrera et al. compared transmyometrial or transvaginal injections (*n* = 8 per group) for establishing orthotopic xenografts using the HEC-1A cell line transfected with luciferase for BLI. In transmyometrially injected mice, 75% developed orthotopic tumors, and all of them had metastatic spread in the pelvic cavity and lymph nodes, compared to 12.5% in the transvaginal group, in which only one mouse developed pelvic metastases. The authors concluded that the transmyometrial procedure is more efficient in generating orthotopic tumors and metastatic spread. To study the metastatic potential and to mimic advanced-stage EC, HEC-1A cells were transmyometrially injected into seventeen nude mice, and tumors were followed with BLI. Developed tumors were detected in 94% of mice, and metastases in the bladder; in the para-aortic lumbar lymph nodes; and in the para-aortic renal, mediastinal, mesenteric, inguinal, and axillary lymph nodes were detected. Furthermore, 80% of mice developed metastases affecting the liver, spleen, pancreas, kidneys, and diaphragm, and in 73% of mice, hematogenous metastases in the lungs were detected. Authors concluded that this model mimics advanced stages of aggressive type-2 EC (p53-positive, hormone receptor-negative, high percentage of Ki67-positive cells) [59]. To study hormonal treatment in EC and to be able to control and modulate steroidal exposure and mimic conditions like post menopause, work from our laboratory (Konings et al.) focused on the generation of an estrogen-dependent orthotopic model of EC using Ishikawa cells stably transfected with BLI. The endogenous source of estrogens was removed by ovariectomy, and exogenous E2 supply was achieved using silicone implants (E2–MedRod^®^) placed in the interscapular region, which provided a constant release of E2 for at least 10 weeks. The orthotopic engraftment take rate was 100%, and E2 was shown to promote tumor growth, lymphovascular (LVI) invasion (in 87% of mice), and metastases (in 100% of mice) in peritoneal and thoracic cavity organs, all features being significantly reduced in placebo–MedRod^®^ mice [60]. Using this model, we tested the efficacy of a novel inhibitor of the enzyme 17β-HSD-1, which catalyzes the last step in the biosynthesis of estradiol converting the weak estrogen estrone (E1) to the potent estrogen 17β-estradiol (E2) (Xanthoulea et al.). MedRod^®^ implants were designed to constantly release a physiologically relevant concentration of E1 (E1–MedRod^®^), which was converted to E2 by the Ishikawa cells expressing the 17β-HSD-1 enzyme. Intratumorally synthesized E2 (since only Ishikawa cells, but not murine cells, were able to convert E1 to E2) promoted tumor growth and metastases (seen in peritoneum, lymphovascular space, and thoracic cavity). Inhibitor-treated mice showed reduced levels of E2 and a significant reduction in both tumor growth (by 65%) and metastases (by 35%), highlighting the importance of intracrinology and of intratumorally produced E2 in driving tumor growth and suggesting a potential for endocrine treatment in EC [61]. Medina-Gutiérrez et al. developed a novel CXCR4-overexpressing orthotopic mouse model to improve metastagenesis to current models. CXCR4 is a chemokine receptor that is upregulated in EC. To develop this model, the authors used the endometrial adenocarcinoma cell line AN3CA transfected with both CXCR4 and luciferase for BLI and injected them in the uterus of NSG mice. The model mimicked the aggressive behavior of advanced-stage EC in humans and showed metastases to all clinically relevant sites, such as the liver, lung, ovaries, peritoneum, and abdominal lymph nodes. The primary tumor and metastases maintained high CXCR4 membrane expression. This model could thereby be used for the future evaluation of therapeutics targeting CXCR4, which will benefit CXCR4+ EC patients who currently lack an effective therapy [62].

In the above studies, the induction of orthotopic tumors was achieved by the intra-uterine injection of cell suspensions of EC cell lines, which, however, lack a three-dimensional tissue architecture and might lead to different biological behavior. Therefore, in some studies, cells from EC cell lines were first injected subcutaneously in mice to generate xenografts, which were subsequently excised, and tumor fragments were transplanted orthotopically in mice. Doll et al. used HEC-1A cells (transfected with the *RUNX1* and *GFP* genes) to generate subcutaneous xenografts in nude mice, and subsequently, 1 mm^3^ fragments of these xenografts were sutured onto the posterior Y-shaped bifurcation of the uterus [63]. After six weeks, the orthotopically grown tumors developed metastases to the para-aortic lymph nodes (75% of mice). Mice with tumors overexpressing RUNX1 developed micro metastases to the lungs (100%), compared to only two out of eight un-transfected tumors and one out of eight tumors transfected with empty vector. The lung lesions showed similar histological features to the original tumor, and the authors concluded that RUNX1 is an inducer of distant metastases, and the model represents advanced-stage endometrial cancer with lymph node metastasis (stage IIIC) and distant metastases (stage IVB). Comparably, Taurin et al. generated subcutaneous xenografts of AN3CA cells in NOD/SCID mice. Fragments of these tumors were again subcutaneously injected into nude mice for further amplification. Tumor fragments of 1–2 mm^3^ were subsequently orthotopically implanted in nude mice. The authors evaluated the efficacy of a novel small tyrosine kinase inhibitor, AL3818 (which targets different proteins among which FGFR2), alone or in combination with conventional carboplatin and paclitaxel. The AN3CA tumors were characterized by the high expression of a mutant FGFR2 protein that is constitutively active. AL3818 significantly reduced tumor volume compared to chemotherapy alone, but with no superior effect when AL3818 was combined with chemotherapy [64]. Estrogen-independent ECs may be sensitive to increased levels of luteinizing hormone (LH), due to the elevated expression of luteinizing hormone receptor (LH-R) in primary EC, which is a characteristic of post menopause. To define the role of LH-R, Pillozzi et al. established subcutaneous xenografts of LH-R-transfected HEC-1A cells in nude mice and subsequently orthotopically sutured 1 mm^3^ of the subcutaneously grown tumors onto the posterior face of the uterus in 27 nude mice. Mice were treated daily with LH to mimic the condition of menopause. The total engraftment rate was 93%, and LH-R overexpressing mice developed metastases to the lymph nodes (100%), bladder (78%), spleen (22%), diaphragm (33%), and lungs (100%), with lymph node invasion being significantly higher in the LH-R-overexpressing mice compared to controls. The authors concluded that the overexpression of LH-R increased local invasion and metastatic spread, and its detection may serve as a diagnostic tool to identify high-risk EC patients [65].

### 4.2. Patient-Derived Orthotopic Xenograft Models

Cabrera et al. were the first in 2012 to develop a PDTX model using two patient grade-2 endometrioid carcinomas. To amplify the tumoral volume, a 1 mm^3^ tumor fragment was collected after surgery and subcutaneously injected into the subscapular region in nude mice. From these xenografts, a fragment of 1–1.5 mm^3^ was mechanically dissociated and injected transmyometrially into the uteri of nude mice. Tumors developed in 90% of mice with a histological pattern similar to the original patient tumor. All mice showed myometrial infiltration, which developed from the inside to the outside of the uterus, as is also observed in patients. Abdominal metastases developed in 66% of the mice, and 77% had dissemination in the pelvic cavity. Mice also developed hemorrhagic ascites, and one mouse showed lymph node metastasis. No hematogenous metastases were observed. The xenografts maintained the molecular and histological characteristics of the original tumors, reproducing glandular patterns and expressing hormone receptors, thus indicating that the model is suitable for preclinical studies [59]. Eritja et al. used three previously established orthotopic xenografts (grades 1–3), re-implanted small pieces into the uterus of nude mice, and randomized them into four treatment groups: placebo, sorafenib, chloroquine (CQ; to inhibit autophagy), and sorafenib plus chloroquine. Sorafenib–CQ markedly impaired tumorigenesis when compared with either condition alone, showing the potential of autophagy inhibition as a complementary therapy to sorafenib in advanced or recurrent EC [30]. Jeong et al. established three orthotopic PDX models of three different sets of ECs, consisting of grade-1, -2, and -3 tumors. Nude mice were first given a daily subcutaneous (0.1 μg for 3 days) or intraperitoneal injection (1 μg for 5 days) of 17β-estradiol or vehicle. Tumor tissue samples were cut into 2–3 mm small fragments and pushed into the lumen of the uterine horn of nude mice. All three tumors were successfully engrafted with an engraftment rate of 100%. However, none of the xenografts developed metastasis [50]. Shen et al. aimed to develop a PDTX model that recapitulates the effects of a high-fat diet on EC, as one of the major risk factors for EC is obesity. The authors first subcutaneously implanted 1 mm^3^ tumor fragments of ER-positive endometrioid adenocarcinomas in nude mice to amplify the tumor (with an observed 66% take rate). Subsequently, 1 mm^3^ subcutaneous xenografts were implanted in the uterine cavity at the level of the fundus of 20 nude mice (with an observed 90% take rate). Mice were given a normal or a high-fat diet to investigate the effects on tumor growth. The authors monitored tumor growth by ultrasound, using a small animal ultrasound imaging platform. In 65% of mice, the adhesion of the tumor to the peri-uterine tissue was observed, and in 40% of mice, the formation of suspected satellite metastases around the tumor was detected. The orthotopic tumors showed similar histomorphology compared to the original tumors and ER expression. A high-fat diet significantly promoted tumor growth by upregulating genes in the estrogen signaling pathway and by increasing ER protein expression [54].

### 4.3. Patient-Derived Organoids for Establishing Orthotopic Xenografts

Two studies used organoids established from patient tumors to generate orthotopic xenografts. Espedal et al. first established organoids from grade-3 endometrioid EC and subsequently (at passage 14) injected them into the uterine horn of NSG mice. After three weeks, all mice developed a primary tumor. Tumor progression was monitored with MRI and dynamic ^18^F-fluorodeoxyglucose (FDG)-PET imaging to detect the tumor size and metabolism. A sub-cohort of mice were further included in a treatment study with standard cytotoxic chemotherapy (PTX-CBP). Larger tumors had higher metabolic activity, and PTX-CBP-treated tumors had lower volumes and less metabolic activity, indicating that advanced MRI and PET imaging methods allow for the non-invasive and quantitative monitoring of tumor progression and treatment response in preclinical EC models. This study was the first in using dynamic ^18^F-FDG-PET and MRI imaging in an orthotopic PDOX EC model [23]. Furthermore, Berg et al. developed organoid-based orthotopic xenografts from grade-1–3, non-endometrioid serous and clear cell EC patient tumors in NSG mice, with an engraftment rate of 80%. Tumor growth was monitored by in vivo small animal imaging, using near-infrared fluorescent (NIRF) imaging or magnetic resonance imaging (MRI). Metastatic lesions were also detected in sites that are commonly seen in endometrial cancer patients, like the ovaries, kidney, pancreas, liver, diaphragm, and pelvic and renal lymph nodes. Xenografts were shown to mimic the tissue architecture, protein biomarker expression, and genetic profile of the original tissue and to exhibit individual responses to conventional PTX-CBP chemotherapy that reproduced the corresponding organoid responses in vitro [24].

**Table 2:** Orthotopic xenograft EC models and their metastatic outcomes.

	Intrauterine	Metastases
**CDXs**	Kamat et al. *Clin Cancer Res* (2007) [55]	Cell suspension of Ishikawa and HEC-1A. Mets: peritoneum, kidney, mesentery, liver, lymph nodes.
	Doll et al. *Int J Cancer* (2009) [63] #	Tumor fragments of HEC-1A-derived s.c. xenografts sutured onto the posterior face of the uterus. Mets: lymph nodes, lungs.
	Lee et al. *Clin Cancer Res* (2010) [56]	Cell suspensions of Ishikawa and HEC-1A. Mets: abdominal cavity, pelvis, mesentery, omentum, liver, porta hepatis, lymph nodes, diaphragm.
	Takahashi et al. *Cancer Lett* (2011) [57]	Cell suspension of Ishikawa. Mets: not reported.
	Takahashi et al. *Cancer Sci* (2011) [58]	Cell suspension of HEC-1A. Mets: lymph nodes.
	Cabrera et al. *Clin Exp Metastasis* (2012) [59]	Cell suspension of HEC-1A. Mets: bladder, perivesical fat, lymph nodes, liver, spleen, pancreas, kidney, diaphragm, lungs.
	Pillozzi et al. *Front Oncol* (2013) [65] #	Tumor fragments of HEC-1A-derived s.c. xenografts sutured onto the posterior face of the uterus. Mets: lymph nodes, bladder, spleen, diaphragm, lungs.
	Haldorsen et al. *PLoS One* (2015) [52]	Cell suspension of Ishikawa. Mets: ovaries, kidney, spleen, pancreas, liver, connective tissue, lymph nodes, adrenal glands, lungs.
	Taurin et al. *Int J Gynecol Cancer* (2018) [64] #	Tumor fragments of AN3CA-derived s.c. xenografts. Mets: not reported.
	Konings et al. *Int J Mol Sci* (2018) [60]	Cell suspension of Ishikawa. Mets: LVI, abdominal (liver, intestine, spleen, stomach, kidney), lungs.
	Fonnes et al. *Cancers* (2020) [53]	Cell suspension of Ishikawa and HEC1B. Mets: abdominal, pancreas, lungs.
	Xanthoulea et al. *Cancer Lett* (2021) [61]	Cell suspension of Ishikawa. Mets: LVI, abdominal, lungs.
	Medina-Gutierrez et al. *Biomedicines* (2022) [62]	Cell suspension of AN3CA. Mets: liver, ovaries, peritoneum, abdominal lymph nodes, lungs.
**PDTXs**	Cabrera et al. *Clin Exp Metastasis* (2012) [59] #	Cell suspension of patient-derived s.c. amplified xenografts. Mets: abdomen, pelvic cavity, lymph nodes.
	Haldorsen et al. *PLoS One* (2015) [52] *	Cell suspension of primary tumor. Mets: not reported.
	Eritja et al. *Autophagy* (2017) [30] ^	Tumor fragments of patient-derived i.u. amplified xenografts. Mets: not reported.
	Fonnes et al. *Cancers* (2020) [53] *	Cell suspension of primary tumor. Mets: not reported.
	Jeong et al. *Gynecologic Oncology* (2021) [50]	Patient-derived tumor fragments. Mets: no metastases observed.
	Shen et al. *Sci Rep* (2023) [54] #	Tumor fragments of patient-derived s.c. amplified xenografts. Mets: suspected satellite metastases around the tumor.
**PDOXs**	Berg et al. *Communications Medicine* (2021) [24]	Injection of organoids. Mets: ovaries, kidneys, pancreas, liver, diaphragm, lymph nodes.
	Espedal et al. *J Transl Med* (2021) [23]	Injection of organoids. Mets: not reported.

* CDXs using cell lines generated in parallel experiments. # Initial subcutaneous tumor generation from either cell lines or patient tumor fragments, and subsequent orthotopic implantation. ^ Initial intrauterine amplification step and subsequent intrauterine implantation.

## 5. Discussion

### 5.1. Comparison of Different Xenograft Locations

In the present review, we provide a detailed overview of the different xenograft mouse models that have been employed to study EC. Subcutaneous xenografts generated using cell lines, patient-derived tumor tissue, and lately, patient-derived organoids have been most widely used because of technical convenience. Subcutaneous xenografts are easy to establish, tumors grow fast and to large volumes, and their growth can be easily monitored using simple tools (palpation, caliper) and without the need for imaging techniques. These make them convenient and fast models to easily test drug responses. Nonetheless, subcutaneous xenografts have low engraftment rates (40–60%, depending on tumor histology and grade, with higher engraftment rates for higher-grade and more aggressive subtypes), they lack the complex tumor microenvironment and interactions present in the original tissue, and they do not present metastases. Therefore, these have been gradually replaced in recent years by more physiologically relevant models. A number of studies were performed on xenografts established in the subrenal capsule of mice. These models seem to achieve better engraftment rates compared to subcutaneous models, although in a direct comparison by Zhu et al. [46], differences did not reach significance (50% vs. 62.5% for subcutaneous and subrenal capsule xenografts, respectively). In addition, these models can develop local metastases to peritoneal organs, which depend on the individual tumor characteristics [48]. However, comparably to subcutaneous grafts, these models do not grow in the uterine microenvironment, which limits their translational power. Moreover, tumors have relatively slow and constrained growth: in a direct comparison, F0-F1 engraftment time was 4–5 weeks for subcutaneous vs. 8–9 weeks for subrenal capsule xenografts, respectively [46]. Moreover, imaging methods are necessary to monitor xenograft development. To increase the translational potential and clinical relevance, orthotopic EC models have been developed, which can recreate a physiologically relevant tumor microenvironment, tissue-specific architecture, and vasculature. Orthotopic EC models have very high engraftment rates (around 100%) and, most importantly, show metastases to clinically relevant sites. Metastatic spread in EC patients is seen primarily to the pelvic cavity, the peritoneum, the pelvic and para-aortic lymph nodes, and the adnexa [66]. Some of the orthotopic models reviewed earlier developed pulmonary metastases, which occur via the hematogenous/lymphatic routes. Pulmonary metastases have been reported as the most common distant metastases stemming from EC in humans [67]. Overall, orthotopic models capture key events of human EC progression, from initial tumor growth and myometrial infiltration, which develops from the inside to the outside of the uterus, to metastatic spread to clinically relevant sites, thus making them highly relevant for translational purposes. The disadvantages of orthotopic models are the requirement of complex microsurgical skills to establish intrauterine tumors and the difficulty to monitor tumor growth, for which in vivo imaging technologies are required. Different imaging modalities have been developed and are being optimized, such as BLI, static and dynamic PET-CT, MRI, and NIRF, but all require complex/expensive dedicated devices. An additional point of reflection in cases where intrauterine injections of cancer cell suspensions are used to establish orthotopic tumors is the possibility that small amounts of cellular solution may leak into the abdominal cavity from the injection site. This may result in the development of foci of growing tumor cells in the abdominal cavity, resembling metastases. As shown in Table 2, in most studies employing orthotopic injections of cell suspensions of either EC cell lines, dissociated tumors, or organoids, local metastases to peritoneal organs are reported. Four studies used tumors fragments inserted into the uterine horns. Two studies give no information regarding metastases [30,64], one study reports suspected satellite metastases around the tumor [54], while interestingly, Jeong at al. report a 100% engraftment rate but no metastases using three different PDTXs [50]. Two studies using tumor fragments from HEC-1A-derived subcutaneous xenografts report metastases. However, fragments appear to have been sutured onto the external posterior face of the uterus [63,65]. Therefore, histologically assessing the infiltration (and not simply the adhesion on the surface) of cancer cells in peritoneal organs/structures can be more informative regarding local metastases. Moreover, metastases to distant locations such as the lungs, only reached via hematogenous/lymphatic routes, or the assessment of lymphovascular space invasion, might be more informative regarding the metastatic potential of the model. In Table 3, we summarize the advantages and disadvantages of the most common implantation sites used to develop EC xenograft mouse models.

### 5.2. Cell Lines Compared to Patient-Derived Material and Factors Influencing Model Establishment

Various source materials can be used to generate EC xenograft models, like established cell lines, patient-derived tumor fragments, or organoids generated from patient tumors, each presenting advantages and disadvantages. Established EC cell lines [68] have been extensively used since these are readily available, low-cost, and easy to grow, and they are immortalized; hence, they can be maintained for a high number (theoretically unlimited) of generations. Cell lines can also be easily genetically manipulated to express a reporter gene for in vivo non-invasive growth monitoring of xenografts, but also for the expression of a specific drug target, or an oncogene/tumor suppressor gene, or for loss-of-function studies. However, since these cell lines are immortalized and heavily propagated in vitro, they may have accumulated mutations and aberrations that have altered their genetic make-up and behavior compared to their original tumor. Cell lines also represent only a small number of human tumors and therefore fail to recapitulate both the large diversity of cancers occurring in humans, but also the cellular and molecular heterogeneity of the individual tumors. Therefore, when using cell lines, a few recommendations need to be followed, including the purchase of new stocks from repositories and the maintenance of early-passage cultures to avoid the use of heavily propagated cells. Moreover, it is important to ensure cell line authenticity by STR profiling, since the widespread use of cell lines over recent decades might have led to misidentification or cross-contamination with unrelated cell lines, which can cause important biases in research outcomes [69]. Patient-derived models (PDTXs and PDOXs) are less widely available than cell lines and present several challenges for their establishment and growth. Due to the limited amount of patient tumor, PDTXs usually require time-consuming amplification steps in a number of mouse generations before being established and used in preclinical studies [37]. PD(T/O)X platforms established through academic collaborations or by commercial companies nowadays offer models derived from various tumor subtypes, thus circumventing the limitations related to the high costs/time required to establish and maintain these models in house [70]. Moreover, it is also more complex to manipulate genetically primary cells and introduce transgenes or induce targeted genetic alterations. However, these models recapitulate the cellular heterogeneity and characteristics of the patient tumor and can accurately capture patient responses to therapies. Most studies using EC PDTX or PDOX models showed good maintenance of the histopathological features and similar mutational profiles compared to the original tumors, with minimal accumulation of CNA events [22,24,37,41,42,47,48,50,59]. Hence, PDTXs and PDOXs have strong potential in the era of personalized medicine. Currently, the major limitation in implementing these models to guide patient care is the long time required to establish and test the models (see also later, limitations). The introduction of novel technologies such as, for example, miniPDXs, which substantially speed up the xenografting process [71], can help in the realistic application of xenografts for personalized treatments. There is also growing interest in utilizing patient-derived organoids (PDOXs) to generate xenograft models since, like PDTXs, these are representative of individual patient tumors, but they are easier and more cost-effective to establish, allow for genetic modifications, and are convenient for high-throughput drug screening. Pauli et al., Berg et al., and Espedal et al. generated organoid-based subcutaneous and intrauterine EC xenografts [22,23,24]. They observed high engraftment rates, the maintenance of histopathological features, and excellent concordance in the genetic profiles and protein biomarker expression between the native tumor and both organoids and PDOXs. PDOXs exhibited individual responses to chemotherapy, and responses were concordant between PDOXs and in vitro organoid models. In a recent publication, Sengal et al. [44] generated organoids from previously established PDTX EC models [41] and showed that these PDTX-derived organoids (PDXOs) are stable and able to retain the morphological and molecular characteristics of parental tumors after cryopreservation and/or serial passaging (P15). Thus, PDXOs from already established PDTX lines are a valuable resource for high-throughput drug screening and research.

### 5.3. Translational Potential of Xenograft Models, Limitations, and Recommendations

Although the PDTX and PDOX EC models were shown to represent the original characteristics of the tumor fragment they derive from with high fidelity, these models do not necessarily capture the intra-tumor heterogeneity of the entire tumor, and most studies have not examined if isolated biopsies sufficiently represent the whole tumor. This is an important aspect to be considered, since intra-tumor heterogeneity could potentially impact treatment responses. To increase the clinical relevance of these models and to better capture the entire landscape of tumor heterogeneity, multiple fragments of the primary cancer could be used to generate different lines of xenografts for a single tumor. However, this method is still very subjective; for instance, in defining the number of sufficient fragments and the part of the tumor, these should be isolated. An alternative strategy could be to establish models from the regions of the tumor that are most likely responsible for clinical outcomes, for example, from the tumor invasion front, from metastatic lesions, or from circulating tumor cells, as shown already for a number of cancers [72,73]. Another important challenge of these models is the fact that components of the tumor microenvironment, such as stromal or immune cells, are not adequately represented. As shown in the above studies, human stroma is gradually replaced by murine stroma during in vivo xenograft passaging [37,50], while in some cases, carcinosarcoma-derived PDTXs exhibited a loss of the epithelial component and prevalence of the mesenchymal part, and other carcinosarcomas maintained only the epithelial component [43,50]. A novel technology that can help circumvent the absence of an immune component in xenografts is the development of humanized immune system mice, which allow us to study tumor–immune interactions and to test the effect of immunotherapies, without host rejection. For endometrial cancer, no reports using humanized mice have been published yet.

### 5.4. Conclusions

Xenograft models and, in particular, patient-derived models are important tools that recapitulate well the general characteristics of parental tumors and can be used to assess patient responsiveness to drugs, thus aiding the development of personalized treatment approaches. Due to the timeline needed to establish these models, for the moment, such information can mainly regard second-line treatments. The largest unmet needs in EC are the management of NSMP and p53 abn subtypes. Women with p53 abn cancers have poor prognosis and few therapeutic (targeted) options. NSMP represents the largest molecular subgroup and includes a very heterogenous group of patients with variable prognosis and need for better classification and prediction of drug response. Future research should enlighten what therapeutic mechanisms could be exploited in these patients or in subgroups of them.

## Figures and Tables

**Table 3:** Advantages and disadvantages of the most common implantation sites used in heterotopic or orthotopic EC xenograft mouse models.

	Advantages	Disadvantages
**Subcutaneous**	Technically easy to perform	Non physiological location/poor translational value
	Easy to monitor tumor growth	Low engraftment rates
	Fast tumor growth	No metastases
	Cost and time effective	
	Tumors can grow to large volumes	
**Subrenal capsule**	Improved engraftment rates compared to subcutaneous models	Non physiological location/poor translational value
	Regional metastases (peritoneum, liver) reported in some studies	Difficult to monitor tumor growth (need for in vivo imaging techniques/probes)
		Slow and constrained tumor growth
**Orthotopic**	Highly relevant due to physiological location	Technically difficult microsurgical skills required
	High engraftment rates	Difficult to monitor tumor growth (need for in vivo imaging techniques/probes)
	Regional spread (uterus, pancreas, peritoneum, spleen, liver)	Potential leakage of tumors cells from the injection site in the abdominal cavity (in case a tumor cell suspension is used)
	Lymphatic and hematogenous metastases	

## Supplementary Materials

The following supporting information can be downloaded at: https://www.mdpi.com/article/10.3390/cancers16233994/s1, Table S1: Cell-lines and resource identifiers (RRIDs).
