# Supplementary material for: Murine Xenograft Models as Preclinical Tools in Endometrial Cancer Research
Source: Cancers (Basel). 2024 Nov 28;16(23):3994. doi: 10.3390/cancers16233994 (PMC11640439; doi:10.3390/cancers16233994)
Supplement: Supplementary file 1 [file cancers-16-03994-s001.zip › cancers-3289876-supplementary.pdf]

Supplementary Table S1. Cell-lines and resource identifiers (RRIDs)

	subcutaneous	*cell-lines and resource identifiers (RRIDs)*
CDXs	Dowdy et al. *Mol Cancer Ther* (2006) [25]	Ark2 (RRID:CVCL_IV73)
	Takahashi et al. *Int J Oncol* (2009) [26]	HEC-1A (RRID:CVCL_0293)
	Pant et al. *PLoS One* (2012)[27]	Ishikawa (RRID:CVCL_2529)
	Packer et al. *Mol Cancer Ther* (2017) [28]	AN3CA (RRID:CVCL_0028) JHUEM2 (RRID:CVCL_4656)
	Eritja et al. *Autophagy* (2017) [30]	MFE-296 (RRID:CVCL_1406)
	Packer et al. *Mol Oncol* (2019) [29]	AN3CA (RRID:CVCL_0028)
PDTXs	Groeneweg et al. *Gynecol Oncol* (2014) * [31]	ARK1 (RRID:CVCL_IV72) ARK2 (RRID:CVCL_IV73) SPEC2 (RRID:CVCL_A679)
	Groeneweg et al. *Clin Cancer Res* (2014) * [32]	ARK2 (RRID:CVCL_IV73) SPEC2 (RRID:CVCL_A679)
	Bradford et al. *Gynecol Oncol* (2014) # [33]	
	Depreeuw et al. *Gynecol Oncol* (2015) [37]	
	Dosil et al. *J Pathol* (2017) * [34]	HEC-1A (RRID:CVCL_0293) MFE-296 (RRID:CVCL_1406)
	Yu et al. *Anticancer Drugs* (2017) * [35]	AN3CA (RRID:CVCL_0028)
	Cuppens et al. *Gynecologic Oncology* (2017) [38]	
	Cuppens et al. *Clinical Cancer Research* (2017) [39]	
	Zhu et al. *Int J Gynecol Cancer* (2018) [46]	
	Felip et al. *Gynecol Oncol* (2019) *[36]	HEC-1A (RRID:CVCL_0293)
	Shin et al. *Cancers (Basel)* (2022) [40]	
	Bonazzi et al. *Genome Med* (2022) [41]	
	Villafranca-Magdalena et al. *International Journal of Molecular Sciences* (2022) [42]	
	Imai et al. *Scientific Reports* (2023) [43]	
	Sengal et al. *npj Precision Oncology* (2023) [44]	
PDOXs	Pauli et al. *Cancer Discov* (2017) [22]	

* Tumor subtype matched CDXs generated in parallel experiments.

# Initial subcutaneous propagation step of patient tumor.
